# The therapeutic revolution in thyroid eye disease: from orbital radiotherapy to teprotumumab and AI

**DOI:** 10.3389/fmed.2026.1758015

**Published:** 2026-02-05

**Authors:** XiaoLi Yuan, Han Li, Feng Wang

**Affiliations:** Department of Head and Neck Tumor Multimodality Treatment, Cancer Center, West China Hospital, Sichuan University, Chengdu, Sichuan, China

**Keywords:** artificial intelligence, biological therapy, IGF-1R, IL-6, orbital fibroblasts, teprotumumab, thyroid eye disease, TNF-α

## Abstract

Thyroid eye disease (TED) is a vision-threatening and quality-of-life-impairing manifestation of autoimmune thyroid disease, driven by orbital fibroblast activation, inflammation, and tissue remodeling. This review synthesizes current evidence on TED epidemiology and pathogenesis, with a particular focus on the pathogenic synergy between the thyrotropin receptor (TSHR) and the insulin-like growth factor-1 receptor (IGF-1R). We discuss how this receptor complex propagates intracellular signaling that leads to disease hallmarks: fibroblast proliferation, glycosaminoglycan secretion, and adipogenesis. While we outline the established paradigm of management—encompassing glucocorticoids, orbital radiotherapy, and surgery—a key emphasis is placed on the recent therapeutic revolution ushered in by targeted biological agents, most notably IGF-1R inhibition. As well as research on new targets for immunotherapy such as Tregs and other aspects such as IL-6 or TNF-α. Finally, we explore the nascent role of artificial intelligence in refining diagnosis and prognostic assessment. This overview aims to equip clinicians and researchers with a forward-looking perspective on the evolving landscape of TED management.

## 1 Introduction

Thyroid Toxic Ophthalmopathy (TTO), also known as thyroid eye disease (TED), is an organ-specific autoimmune disease whose development and progression are closely associated with thyroid dysfunction. Statistics indicate that toxic thyroid eye disease ranks among the leading causes of orbital disorders in adults, accounting for approximately 20% of cases. It represents the most common extra-thyroid manifestation of Graves’ disease (GD), occurring in 25%–40% of GD patients. The condition may also be observed in a minority of individuals with chronic lymphocytic thyroiditis, hypothyroidism, and even in those with normal thyroid function (1).

The core pathological features of TED involve inflammatory infiltration of orbital tissues (predominantly mononuclear cells) and fibroblast activation. Its clinical manifestations are diverse, ranging from functional impairments such as proptosis, diplopia, and decreased visual acuity to optic nerve compression leading to blindness. Concurrently, the characteristic facial changes and visual impairment inflict significant psychological stress and emotional distress on patients, severely compromising their quality of life (2).

Thyroid eye disease typically progresses through an initial inflammatory (active) phase followed by a stable period, during which activity ceases for approximately 18–24 months (entering an inactive or “exhausted” state). Mild to moderate cases often resolve spontaneously, while severe TED requires therapeutic intervention. Treatment generally focuses on symptom relief, with complete recovery to normal function being highly unlikely (3). Early diagnosis, control, and elimination of modifiable risk factors, along with early intervention for mild TED, can effectively reduce the risk of progression to more severe forms of the disease while significantly improving patients’ quality of life. In recent years, with in-depth research across various disciplines, new advances have been made in TED’s pathogenesis, diagnostic methods, and treatment approaches.

## 2 Epidemiology

According to data from the 2017 European Group on Graves’ Ophthalmopathy (EUGOGO), the annual incidence rates in Europe and Japan are 10/10,000 and 16/10,000, respectively. Approximately 5% of GD patients exhibit significant TED symptom (4). The prevalence of Graves’ disease (GD) among Chinese adults is 1.2%, while the prevalence of TED is approximately 300 per 100,000. The disease burden in the Chinese population is relatively high (1). Research indicates that the prevalence of TED does not differ significantly across racial groups, ranging from approximately 90 to 300 cases per 100,000 people (1). Research also indicates that the age of onset for Graves’ disease exhibits a bimodal distribution: among female patients, the peaks occur at 40–44 and 60–64 years; among male patients, the peaks occur at 45–49 and 65–69 years (5). Age is a significant factor influencing the severity of TED, with the disease often presenting more severely in elderly patients. Additionally, men tend to experience more severe TED as they age (6).

The occurrence and progression of TED result from the combined effects of multiple risk factors against a background of genetic susceptibility. Numerous studies indicate that smoking increases the risk of ocular involvement in GD patients, making it the most significant exogenous factor contributing to the development and worsening of TED (7, 8). The expression level of the thyroid-stimulating hormone receptor (TSHR) is often correlated with the severity of Graves’ disease, and patients with severe thyrotoxicosis typically exhibit elevated levels of thyrotropin receptor antibodies (TRAb) in their serum (9). Radioactive iodine may exacerbate the clinical symptoms of TED. This exacerbation may be temporary and could be attributed to a surge in autoimmune responses following radioactive iodine therapy (10). Physical damage to the eye socket may also be associated with the onset and exacerbation of TED -related inflammation (11). Research findings on the gut microbiota and TED indicate that differences in immune responses and alterations in thyroid function may be associated with the heterogeneity of induced immune responses resulting from changes in the gut microbiota (12, 13). The pathogenesis of hypothyroidism is associated with bacterial overgrowth; however, bacteria fermenting carbohydrates in the gut do not affect thyroid hormone levels (14).

## 3 Pathogenesis

The pathogenesis of TED is complex and its specific mechanisms remain unclear. It is currently widely recognized as an autoimmune disorder resulting from the combined effects of genetic and environmental factors. The disease primarily involves a series of orbital inflammatory reactions mediated by autoantigens and cellular immunity, leading to fibrosis of the extraocular muscles and proliferation of local adipocytes. These changes cause clinical manifestations of thyroid eye disease, such as proptosis (15, 16).

The core mechanism lies in the uncontrolled activation and proliferation of orbital fibroblasts (OFs). This uncontrolled activation and proliferation of orbital fibroblasts is driven by chemotaxis within a pro-inflammatory microenvironment formed by various autoantibodies—such as those targeting the thyroid-stimulating hormone receptor (TSHR) and insulin-like growth factor-1 receptor (IGF-1R)—alongside immune cells including T cells, B cells, and macrophages (16–18). Activated fibroblasts can differentiate into two distinct phenotypes: lipid-rich adipocytes or scar-forming myofibroblasts. This differentiation leads to lipogenesis, which is associated with proptosis and orbital fat expansion. It also involves the production of glycosaminoglycans, hyaluronic acid, and proteoglycans—all hallmarks of scar formation (19). Among the key molecular pathways involved in the activation of orbital fibroblasts, immune dysregulation is the most significant factor (20).

### 3.1 Immune mechanism

#### 3.1.1 TSHR

Thyroid-stimulating hormone receptor is one of the primary autoantigens involved in the pathogenesis of TED (15). TSHR expression levels are elevated in the thyroid and orbital tissues of TED patients, with a more pronounced increase observed in patients during the clinically active phase (21, 22). *In vitro* culture experiments revealed that TSHR expression levels were low in both orbital fibroblasts and normal orbital adipose tissue (23–25). When a patient’s immune balance is disrupted, the high expression of TSHR in orbital tissues makes it a key target for autoimmune attacks. When the immune system produces antibodies against TSHR (TRAb), these antibodies not only act on the thyroid, causing thyroid dysfunction, but also bind to TSHR on the surface of orbital fibroblasts. This binding activates a series of inflammatory signaling pathways, leading to the uncontrolled activation of OFs (16).

Thyroid-stimulating hormone receptor is also a major G protein-coupled transmembrane receptor closely associated with cell proliferation (26). Woeller et al. performed *in vitro* stimulation with thyroid-stimulating hormone (TSH) on orbital fibroblasts from patients with and without thyrotoxicosis. Compared to orbital fibroblasts from the non-thyrotoxicosis group, those from thyrotoxicosis patients exhibited significantly higher proliferative capacity in response to TSH (27). TSH-induced proliferation depends on TSHR expression and requires involvement of the PI3K/Akt signaling pathway. Activation of TSHR stimulates the expression of miR-146a and miR-155. TED-induced fibroblasts produced significantly higher levels of miR-146a and miR-155 compared to the non-TED group. TSH suppresses the expression of its target molecules ZNRF3 and PTEN by upregulating miR-146a and miR-155, thereby releasing the restriction on cell proliferation. This leads to excessive proliferation of orbital fibroblasts, contributing to the pathogenesis of Graves’ ophthalmopathy. Activation of the PI3K pathway in orbital fibroblasts leads to cyclic adenosine monophosphate (cAMP) production and subsequent secretion of glycosaminoglycans (GAGs) such as hyaluronic acid. Uncontrolled activation causes abnormal GAG accumulation, resulting in periorbital and retrobulbar tissue edema. This manifests clinically as exophthalmos and eyelid swelling (28).

#### 3.1.2 IGF-1R

IGF-1R is a tyrosine kinase receptor, a type I transmembrane protein composed of multiple amino acids, widely expressed on the surfaces of various cell types. In orbital fibroblasts, T cells, and B cells from TED patients, IGF-IR expression levels are elevated compared to normal levels (29). IGF-1R participates in cellular growth, proliferation, and anti-apoptotic processes through multiple signaling pathways and molecular mechanisms, playing a key role in the pathogenesis of TED (30).

IGF-1R and TSHR form a complex on the surface of orbital fibroblasts, enabling IGF-1R to participate in TSHR-mediated signal transduction processes (31). The signal transduction processes involving IGF-1R primarily involve the synergistic activation of two non-canonical signaling pathways: the MAPK/Erk pathway and the PI3K/Akt/mTOR pathway (29). IGF-1R exhibits specific functional roles in well-differentiated orbital fibroblasts, thyroid epithelial cells, T cells, and B cells:

(1) In orbital fibroblasts, GD-IgG activates the Akt/mTOR pathway via IGF-1R, thereby inducing the synthesis and release of chemokines such as IL-16 and RANTES. This attracts T cells and monocytes to infiltrate orbital tissues, exacerbating local inflammatory responses (29, 32). It can also significantly upregulate the expression of hyaluronan synthases (HAS1, HAS2, HAS3) in orbital fibroblasts, particularly HAS2 and HAS3, and promote the synthesis of hyaluronan precursors via UDP-glucose dehydrogenase (UGDH). Hyaluronic acid possesses high hydrophilicity. Its substantial accumulation within orbital connective tissue leads to tissue edema and volume expansion, constituting one of the primary mechanisms underlying proptosis in TED patients (29, 33, 34). Orbital fibroblasts exhibit heterogeneity and differentiation potential. Among them, the Thy-1 (CD90)-negative cell subpopulation initiates adipogenesis upon activation of the IGF-1R signaling pathway, leading to fat proliferation. Conversely, the Thy-1 (CD90)-positive cell subpopulation activates and differentiates into myofibroblasts under the synergistic stimulation of inflammatory mediators such as TGF-β (35, 36). Activated fibroblasts possess contractile capabilities (expressing alpha-smooth muscle actin, α-SMA) and the capacity to secrete large amounts of extracellular matrix components such as collagen. This leads to scarring of orbital tissues, dysfunction of extraocular muscles, and compression of the optic nerve (29, 34, 37).

(2) In thyroid epithelial cells, the interaction between IGF-1R and TSHR enhances TSH- or TSAb-mediated cell proliferation and thyroid hormone synthesis. IGF-1 itself can also promote thyroid cell growth through autocrine or paracrine mechanisms and amplify the proliferative effects of TSH (38). Additionally, GD-IgG activates inflammatory pathways in thyroid cells via IGF-1R, inducing IL-16 and RANTES expression, which may promote lymphocyte infiltration into the thyroid and contribute to the maintenance and expansion of thyroid autoimmune responses (39).

(3) In T cells, IGF-1R signaling activates the PI3K/Akt pathway, which serves as a potent cell survival signal (40, 41). Akt can phosphorylate and inhibit pro-apoptotic proteins (such as Bad and Caspase-9), thereby preventing T cell apoptosis (41). The abnormal survival of autoreactive T cells—those capable of recognizing thyroid antigens such as TSHR—is crucial for the persistence of autoimmune diseases (42). IGF-1R signaling confers a survival advantage to these cells that should be eliminated, enabling them to persist long-term in the body and drive disease progression (43, 44). Research has revealed that compared to healthy controls, patients with GD exhibit a significantly elevated proportion of CD4+ and CD8+ memory T cells (CD45RO+) expressing IGF-1R in both peripheral blood and orbital infiltrating T cells. *In vitro* experiments confirmed that IGF-1 enhances the proliferation of these T cells and inhibits Fas-mediated apoptosis. The IGF-1R expressed by lymphocytes may support the expansion of memory T cells in GD (43). IGF-1 signaling has been demonstrated to promote the production of Th2 cytokines such as IL-4 and IL-10 while suppressing the function of Th1 cytokines like IFN-γ (45). This may indirectly promote TRAb production by driving T cell responses toward Th2 polarization (46, 47).

(4) In B cells, IGF-1R signaling directly stimulates B cell proliferation and differentiation while enhancing immunoglobulin synthesis (including IgG). This provides proliferative and antibody-producing signals to autoreactive B cells, leading to elevated TRAb levels that in turn continuously stimulate TSHR on thyroid and orbital cells (48). Studies of monozygotic twins (one affected, one unaffected) revealed that the affected twin exhibited significantly higher proportions of IGF-1R+ T cells and B cells compared to the healthy sibling. Research indicates that elevated IGF-1R expression is driven by non-genetic factors—such as environmental triggers and immune activation—and serves as a functional marker of disease activity (49).

IGF-1R acts as an “amplifier” in T cells and B cells of GD patients. By enhancing lymphocyte survival and function, it disrupts immune tolerance, allowing sustained and amplified autoimmune responses against TSHR. This explains why drugs targeting IGF-1R (such as Teprotumumab) not only directly act on orbital fibroblasts but also indirectly reduce autoantibody levels by modulating immune cell function, thereby achieving systemic disease treatment (50–52).

### 3.2 Genetic factors

Genetic factors play a significant role in susceptibility to toxic thyroid eye disease. Certain genetic polymorphisms are associated with an increased risk of developing TED (53, 54). Research indicates that polymorphisms in the CTLA-4 gene influence T-cell activation and regulation, leading to immune imbalance (55). This increases the incidence of TED. A meta-analysis by Zheng et al. indicates that mouse model studies demonstrate PTPN22 plays a key role in regulating adaptive and innate immunity, and specific variants in the human PTPN22 gene have been shown to be significantly associated with enhanced autoimmune responses (56). Furthermore, certain alleles within the HLA gene family, such as HLA-B8 and HLA-DR3, exhibit significantly higher frequencies in TED patients compared to the general population, suggesting a strong association with genetic susceptibility to the disease (6, 57, 58). Gametic mutations in the GSTP1, CYP1A1, and TP53 genes (rather than mutations in GSTT1 and GSTM1) may be associated with susceptibility to smoking-related Graves’ disease (8, 59). These susceptibility genes may increase an individual’s vulnerability to toxic thyroid eye disease by affecting immune cell function, antigen presentation processes, or inflammatory signaling pathways (58, 60).

**FIGURE 1:**
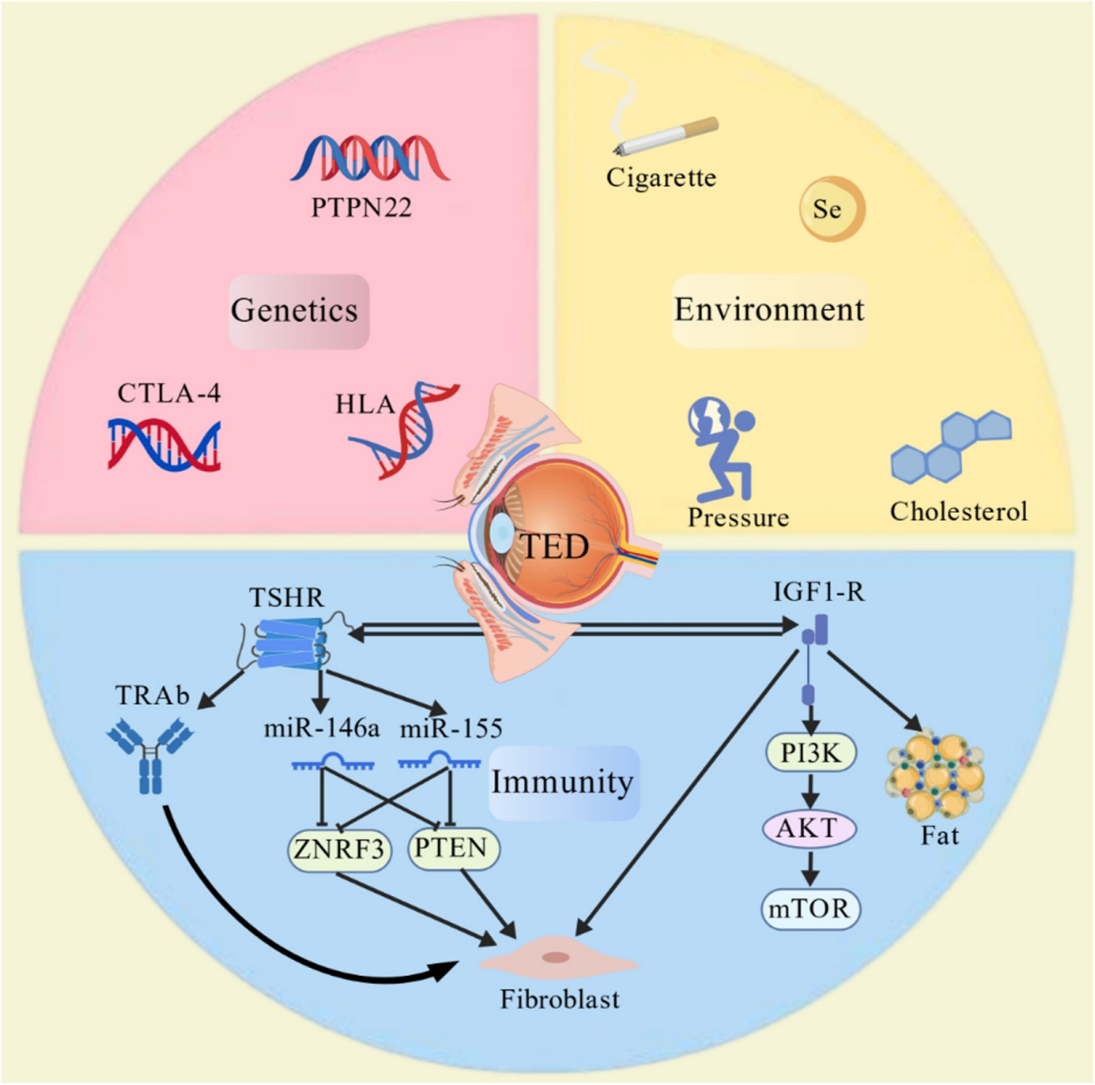
Genetic, environmental, and immunological regulation of thyroid eye disease pathogenesis.

### 3.3 Environmental factors

Environmental factors are significant contributors to the onset of toxic thyroid eye disease. Smoking is a confirmed risk factor, with smokers facing several times the risk of developing the condition compared to non-smokers, and often experiencing more severe symptoms (8, 59). Nicotine, tar, and other components in tobacco can stimulate orbital fibroblasts to express HLA-DR antigens, promote mucopolysaccharide synthesis, and simultaneously reduce the production of soluble interleukin-1 receptor antagonists (sIL-1RA), thereby enhancing the pro-inflammatory effects of IL-1 (61). Additionally, hypercholesterolemia, particularly elevated levels of both low-density lipoprotein (LDL) and high-density lipoprotein (HDL) cholesterol, may increase the risk of disease onset by affecting lipid metabolism and inflammatory responses (62). Retrospective studies indicate that patients with severe TED who receive statin therapy experience a reduced need for reconstructive surgery, indirectly reflecting the impact of hypercholesterolemia on the risk and severity of TED (63). Deficiencies in trace elements, such as selenium, impair the antioxidant defense system, making ocular tissues more susceptible to oxidative stress damage (64–66). Radioactive iodine therapy may induce or exacerbate eye disorders, potentially through increased release of thyroid antigens post-treatment, triggering a more intense immune response (67, 68). Life stressors and pressure may also influence immune function through the neuroendocrine system, acting as triggers for disease (3, 69).

## 4 Clinical manifestations

### 4.1 Ocular symptoms and signs

Clinical manifestations in TED patients are complex and diverse, with common local signs primarily including exophthalmos, diplopia, and ocular swelling. Exophthalmos is typically bilateral but may occur unilaterally, with unilateral cases being more common in individuals with normal thyroid function. Involvement of the extraocular muscles leads to restricted eye movement and diplopia. Conjunctival hyperemia and edema are also frequent symptoms. In severe cases, swollen conjunctiva may protrude beyond the palpebral fissure, causing incomplete eyelid closure and subsequent corneal complications. Compressive optic neuropathy represents the most serious clinical alteration. Thickened extraocular muscles, orbital edema, and elevated orbital pressure compress the optic nerve, leading to decreased visual acuity, visual field defects, or pathological scotomas. Fundus examination reveals optic disk edema or pallor, retinal edema or exudates, and tortuous or dilated retinal veins. Severe cases may progress to optic atrophy and vision loss (70–74).

### 4.2 Systemic symptoms

When toxic thyroid eye disease is accompanied by hyperthyroidism, patients often exhibit systemic symptoms such as tachycardia, restlessness and anxiety, increased appetite, weight loss, and hand tremors. When associated with hypothyroidism, symptoms may include dry skin, memory impairment, emotional detachment, facial edema, and heart failure (15).

### 4.3 Complications

Exposure keratitis, corneal ulcer: Incomplete eyelid closure and protrusion of the eyeball deprive the cornea of eyelid protection, exposing it to air. This leads to corneal dryness and epithelial sloughing, resulting in exposure keratitis. If a secondary infection occurs, a corneal ulcer may form, causing the patient to experience significant pain, photophobia, and tearing (69, 75).

### 4.4 Classification and staging

The severity of the disease is determined based on the NOSPECS (Table 1) grading system proposed by the 2017 edition of EUGOGO and the Clinical Activity Score (CAS) (Table 2) staging system, which guide clinical treatment and efficacy assessment. Commonly used clinical grading methods classify the condition according to the degree of proptosis, involvement of extraocular muscles, corneal involvement, and optic nerve involvement (4).

**TABLE 1:** NOSPECS classification for thyroid eye disease.

Class	Abbreviation and name	Clinical features and signs
0	N: on signs or symptom	No signs or symptoms. There may be abnormal thyroid function.
1	O: only signs	Only signs, no subjective symptoms. Signs include eyelid retraction (the upper eyelid margin is positioned > 2 mm above the corneoscleral limbus) and mild lid lag.
2	S: soft tissue involvement	Soft tissue involvement with manifestations such as eyelid swelling, conjunctival redness, and swelling of the caruncle.
3	P: proptosis	Proptosis (exophthalmos), where the exophthalmometry measurement exceeds the normal range (generally 12–14 mm in adults), with or without symptoms of soft tissue involvement.
4	E: extraocular muscle involvement	Extraocular muscle involvement, manifesting as restricted eye movement, which may cause diplopia (double vision).
5	C: corneal involvement	Corneal involvement, characterized by punctate keratopathy, ulceration, etc., which may lead to decreased vision.
6	S: sight loss (optic nerve involvement)	Sight loss due to compressive optic neuropathy, which can lead to blindness in severe cases.

**TABLE 2:** Clinical Activity Score (CAS) for thyroid eye disease.

Score number	Clinical manifestation	score
1	Spontaneous retrobulbar pain	1 point
2	Pain on eye movement	1 point
3	Eyelid erythema	1 point
4	Conjunctival hyperemia	1 point
5	Conjunctival edema	1 point
6	Swelling of the caruncle	1 point
7	Eyelid swelling	1 point

The Clinical Activity Score (CAS) is commonly used to assess disease activity, comprising seven indicators: spontaneous retrobulbar pain, pain during ocular movement, eyelid hyperemia, conjunctival hyperemia, eyelid edema, conjunctival edema, and recent (within 3 months) increase in proptosis exceeding 2 mm. A CAS score ≥ 3 indicates an active phase. The disease is generally categorized into active and inactive phases. During the active phase, symptoms and signs progress rapidly with marked ocular inflammation, such as eyelid redness and swelling, conjunctival hyperemia, and ocular pain. In the inactive phase, symptoms and signs remain relatively stable with reduced or absent inflammatory manifestations, though ocular morphological changes persist, including proptosis, extraocular muscle hypertrophy, and extraocular muscle fibrosis (76).

## 5 Treatment methods

### 5.1 General treatment

General treatment measures aim to alleviate symptoms and protect the eyes. For patients with incomplete eyelid closure, artificial tears and eye ointments are used to maintain ocular surface moisture, preventing corneal dryness and infection. Wearing sunglasses outdoors reduces exposure to bright light and wind/dust irritation. Elevating the head during sleep alleviates ocular edema. Avoiding prolonged eye use minimizes eye fatigue. Additionally, smoking cessation is a crucial intervention that significantly reduces the risk of disease progression and improves treatment outcomes (69).

### 5.2 Pharmacotherapy

#### 5.2.1 Glucocorticoids

These serve as the first-line treatment for active thyroid-associated ophthalmopathy, acting by suppressing inflammatory responses and immune cell activity. administration may be via oral, intravenous, or local injection routes. The common starting dose for oral prednisone is 40–80 mg daily, gradually tapered based on clinical response. intravenous methylprednisolone pulse therapy is typically administered 1–3 times weekly at 500–1,000 mg per dose for 3–6 consecutive weeks. However, vigilance is required regarding potential side effects such as infection, hypertension, diabetes, and osteoporosis (69, 77).

#### 5.2.2 Immunosuppressants

For patients who are unresponsive to or intolerant of glucocorticoid therapy, immunosuppressants may be considered. Cyclosporine suppresses T-lymphocyte activation and reduces cytokine production. The usual dose is 5–10 mg/kg daily, administered orally in two divided doses. Methotrexate inhibits purine synthesis and interferes with immune cell proliferation. The weekly dose ranges from 7.5 to 25 mg, administered orally or via subcutaneous injection. Close monitoring of blood counts, liver and kidney function, and other relevant indicators is required during immunosuppressant therapy (10).

#### 5.2.3 Biological agents

In recent years, the application of biological agents has introduced new treatment options for thyroid-associated ophthalmopathy. teprotumumab is the first approved monoclonal antibody targeting IGF-1R. By blocking the IGF-1R signaling pathway, it inhibits inflammation and fibrosis, significantly improving ocular symptoms and signs. It demonstrates particularly pronounced efficacy in reducing proptosis and resolving diplopia (78, 79). Linsitinib is a dual small-molecule kinase inhibitor that targets both the IGF-1R and the insulin receptor (IR). It effectively reduces t-cell and macrophage immune infiltration in the orbital tissues of patients with moderate-to-severe TED (80). The phase I clinical trial of K1-70TM, a human monoclonal antibody targeting TSHR, demonstrated favorable safety and tolerability. patients with TED experienced significant improvement in clinical symptoms such as exophthalmos and photosensitivity (81). The current study has a small sample size and requires further expansion to validate its findings. However, as a novel drug, it still holds considerable potential for blocking the action of thyroid-stimulating substances on the TSHR.

### 5.3 Orbital radiation therapy

Irradiating the orbit with high-energy rays suppresses the proliferation of inflammatory cells and the growth of fibrous tissue within the orbit, thereby reducing orbital inflammation and edema. This treatment is suitable for patients in the active phase of the disease who exhibit involvement of the extraocular muscles and compressive optic neuropathy (69). Although the optimal dose remains undetermined, the most common regimen involves administering 20 Gy in 10 fractions of 2 Gy each; however, other regimens may yield superior outcomes. Orbital radiotherapy may induce long-term complications such as radiation cataracts and retinopathy; therefore, it should be used with caution in younger patients and those with contraindications to ocular radiation therapy (82, 83).

Currently, the primary clinical techniques for orbital radiotherapy include the following, which differ in dose distribution, protection of critical organs, and therapeutic efficacy: (1) Three-dimensional conformal radiotherapy (3D-CRT): This involves creating models for fixation and performing thin-slice CT scans to delineate the target volume and critical organs, followed by the design of the radiotherapy plan. Compared to conventional radiotherapy techniques, it reduces damage to surrounding normal tissues to a certain extent (84). However, 3D-CRT still has limitations. For some complexly shaped target volumes, it is difficult to achieve perfect conformal delivery, and protection of critical organs remains suboptimal (82, 85). (2) Intensity-Modulated Radiation Therapy (IMRT): IMRT represents a more advanced form of conformal radiation therapy. By optimizing treatment planning and utilizing devices such as multileaf collimators to modulate radiation intensity, it achieves a more uniform dose distribution within the target area while effectively reducing radiation exposure to surrounding healthy tissues. Studies indicate that in treating thyroid-related eye disease, IMRT significantly reduces radiation exposure to critical organs such as the lens, thereby lowering the risk of complications (86). (3) Volumetric Modulated Arc Therapy (VMAT): VMAT is a technology developed from IMRT. It employs an arc-shaped rotational irradiation method, where the accelerator gantry, collimator, and multileaf collimator move synchronously during treatment to achieve dynamic irradiation of the target area. VMAT offers significant advantages in dose distribution (87, 88).

### 5.4 Surgical treatment

Patients with stable, non-active ocular diseases may opt for surgical intervention to alleviate symptoms. Currently available surgical treatments include: (1) Orbital Decompression Surgery: This procedure involves removing portions of the orbital bone walls to expand the orbital cavity volume, thereby reducing intraorbital pressure. It relieves compression on the optic nerve and extraocular muscles, improving proptosis and ocular symptoms. This is indicated for patients with severe proptosis, compressive optic neuropathy, or corneal exposure that cannot be alleviated by conservative treatment. By removing part of the orbital bone wall, the orbital volume is increased, reducing intraorbital pressure, relieving compression on the optic nerve and extraocular muscles, and improving proptosis and ocular symptoms (89, 90). (2) Extraocular Muscle Surgery: For patients with stable conditions lasting over six months, this procedure restores ocular motility balance and binocular vision function by adjusting the length and attachment points of the extraocular muscles. It is used to correct strabismus and diplopia caused by fibrosis and contracture of the extraocular muscles (91, 92). (3) Eyelid Surgery: Corrective procedures such as levator aponeurosis lengthening and tarsorrhaphy are performed to improve eyelid position and function, thereby protecting the cornea. This treatment is suitable for patients with eyelid retraction and incomplete eyelid closure (93).

## 6 Emerging research directions

### 6.1 New targets for targeted therapy

B lymphocyte stimulator (BLyS), also known as B cell activating factor (BAFF), plays an indispensable role in the activation, survival, and differentiation of B cells. Research by Wang et al. (94) demonstrated that compared to healthy individuals, GD patients exhibited significantly elevated BAFF levels in peripheral serum. Furthermore, expression of the BAFF receptor BR3 on GD patients’ B cells was markedly increased. Following glucocorticoid therapy, serum BAFF levels in patients decreased significantly, and BR3 receptor expression on B cells was also markedly suppressed. Abnormal expression of BAFF and its receptors (particularly BR3) may play a crucial role in the autoimmune process of GD. Restoring the normal expression profile of BAFF receptors could represent a novel therapeutic strategy for treating TED (94).

Key molecules in cytokine signaling pathways also represent highly promising therapeutic targets. Liu et al. demonstrated significantly reduced Epac1 expression in both clinical TED patient samples and orbital tissues from TED mouse models, accompanied by elevated expression of the fibrosis marker vimentin. *In vitro* cellular experiments revealed that Epac1 overexpression suppressed cell proliferation and migration while reducing expression of fibrosis markers including α-SMA, fibronectin, and collagen. It significantly inhibited TGFβ1-induced activation in both normal and TED-affected orbital fibroblasts (OFs), whereas Epac1 knockdown exacerbated TGFβ1-induced fibrosis. Overexpressing Epac1 in the orbital tissue of TED model mice improved TED -like pathological symptoms. Studies indicate Epac1 exerts its anti-fibrotic effect by inhibiting STAT3 phosphorylation. The JAK/STAT pathway inhibitor Stattic effectively reversed the pro-fibrotic effects caused by Epac1 knockdown. As a key negative regulator in TED fibrosis, Epac1 suppresses orbital fibroblast activation by mediating JAK/STAT signaling. This suggests Epac1 may represent a novel therapeutic target for TED. Currently, tofacitinib, a JAK inhibitor, has demonstrated promising efficacy in treating other autoimmune diseases such as rheumatoid arthritis (95).

Tumor Necrosis Factor-α (TNF-α) is also an important potential therapeutic target for TED. As a key pro-inflammatory cytokine, TNF-α levels are significantly elevated in patients with thyroid-associated ophthalmopathy. It activates downstream inflammatory signaling pathways, leading to orbital tissue inflammation, fibrosis, and immune cell infiltration (96). In the field of toxic thyroid eye disease, small-scale clinical trials have explored the use of anti-TNF-α monoclonal antibodies for treatment. Preliminary results indicate that ocular inflammatory symptoms, such as conjunctival hyperemia and edema, have been alleviated in some patients. However, large-scale, multicenter clinical trials are still required to further validate its efficacy and safety (97, 98).

Interleukin-6 (IL-6), as a pro-inflammatory cytokine, shows significantly elevated levels in the serum of patients with active thyroid-induced ophthalmopathy. It activates inflammatory responses in T cells and B cells, leading to tissue proliferation, muscle hypertrophy, and exophthalmos (99). Tocilizumab is a recombinant humanized monoclonal antibody targeting the IL-6 receptor, initially used for treating rheumatoid arthritis and later applied in studies for treating thyroid-associated ophthalmopathy. Studies have demonstrated that tocilizumab reduces memory B-cell concentrations and immunoglobulin levels, alleviates inflammatory tissue reactions in the orbit, and produces varying degrees of improvement in disease activity scores, exophthalmos, TSH receptor antibody levels, ocular motility, diplopia, and visual acuity (100).

### 6.2 Immunomodulatory cell therapy

Regulatory T cells (Tregs) constitute a subset of T cells with immunosuppressive functions, playing an indispensable role in maintaining immune tolerance. In healthy individuals, Tregs effectively suppress the activation and proliferation of autoreactive T cells, thereby preventing the onset of autoimmune diseases. However, extensive research indicates that both the number and function of Tregs exhibit significant abnormalities in patients with thyroid-associated ophthalmopathy (101, 102). Research analyzing peripheral blood and orbital tissue samples from patients revealed that the proportion of Tregs in their peripheral blood was significantly lower than in healthy individuals, with markedly diminished ability to suppress effector T cell function. This allows self-reactive T cells to evade regulatory control, abnormally activate, and attack orbital tissues, triggering inflammatory responses and tissue damage. The diminished immunosuppressive capacity of regulatory T cells may represent a key factor in the pathogenesis of Graves’ ophthalmopathy. Notably, multiple studies have confirmed the potential involvement of Th17 cells in the onset and progression of Graves’ ophthalmopathy, with these cells potentially serving as novel markers of disease activity alongside associated cytokines. Impaired Treg/Th17 balance may play a crucial role in the development of Graves’ ophthalmopathy (103, 104).

Mesenchymal stem cells (MSCs) are a type of multipotent stem cell widely present in tissues such as bone marrow and adipose tissue (105, 106). In terms of immune regulation, MSCs can secrete various cytokines and growth factors through paracrine mechanisms g, such as transforming rowth factor-β (TGF-β) and hepatocyte growth factor (HGF). These factors modulate the activity of immune cells, suppressing the proliferation and activation of T cells and B cells while promoting the generation and functional activity of Tregs (107, 108). Concurrently, MSCs can suppress the release of inflammatory cytokines such as TNF-α and IL-6, thereby alleviating inflammatory responses (109, 110). Human adipose-derived mesenchymal stem cells demonstrate exceptional efficacy in enhancing adipogenesis within orbital tissues in animal models, confirming their feasibility in novel TED therapies. Research confirms that placental mesenchymal stem cells overexpressing PRL-1 significantly inhibit orbital adipogenesis in Graves’ ophthalmopathy by secreting insulin-like growth factor-binding protein, which upregulates FAK phosphorylation and suppresses the PI3K/AKT/mTOR pathway (111). Research confirms that placental mesenchymal stem cells overexpressing PRL-1 significantly inhibit orbital adipogenesis in Graves’ ophthalmopathy by secreting insulin-like growth factor-binding protein, which upregulates FAK phosphorylation and suppresses the PI3K/AKT/mTOR pathway (112).

Research on peripheral blood T lymphocyte subsets provides deeper theoretical support for immunomodulatory cell therapy. Wang et al. found that CD4+ cytotoxic T lymphocyte subsets play a crucial role in the pathogenesis of Graves’ ophthalmopathy and are closely associated with the efficacy of hormone shock therapy. This cell subset exhibits marked clonal expansion and thyroid-stimulating hormone receptor specificity. In patients with Graves’ ophthalmopathy, compared to initial CD4+ T lymphocytes, its diversity is significantly reduced, and pronounced oligoclonal expansion occurs. Upon stimulation by the autoantigen of Graves’ ophthalmopathy—the thyroid-stimulating hormone receptor—CD4+ cytotoxic T lymphocytes become markedly activated and release cytotoxic molecules (113). Hang et al. investigated the effects of rapamycin [an approved mTOR complex 1 (mTORC1) inhibitor] in TED mouse models, *in vitro*, and in refractory TED patients. In the adenovirus-induced model, rapamycin significantly reduced TED incidence, accompanied by decreased CD4+ CTLs and attenuated orbital inflammation, lipoidosis, and fibrosis. CD4+ CTLs from active TED patients exhibited upregulation of the mTOR pathway, while rapamycin reduced their proportion and cytotoxic function. Low-dose rapamycin treatment significantly improved diplopia and clinical activity scores in hormone-resistant TED patients. These findings facilitate precise identification of pathogenic T cell subsets, laying a solid foundation for developing more targeted and effective immunomodulatory cell therapy strategies (114).

In clinical studies of certain traditional Chinese medicines, active components such as phellodendrin, curcumin, puerarin, tannin IIA, and tripterygin have demonstrated inhibitory effects on TED-OF. These compounds hold promise for translation into novel therapeutic strategies (115). A meta-analysis by Li et al. demonstrated that treatment with Tripterygium glycosides can elevate FT3 levels, improve the response rate to TED therapy, and enhance patients’ quality of life (116).

### 6.3 Applications of artificial intelligence in diagnosis and treatment monitoring

In the field of toxic thyroid eye disease, artificial intelligence technology is gradually emerging, offering new approaches and methods for precise diagnosis and treatment monitoring.

In the diagnostic phase, AI can perform in-depth analysis of ocular imaging data (such as CT and MRI scans). One study employed the U-Net deep learning model to automatically segment orbital muscle and fat volumes in orbital CT images. Results demonstrated that machine learning classification models constructed using volume data and metadata achieved high accuracy in distinguishing between idiopathic orbital lesions and those associated with chronic inflammation (117). A deep learning model based on convolutional neural networks (CNN) automatically extracts structural features such as orbital tissues and extraocular muscles in patients with TED. This enables early, accurate, and rapid identification of TED patients, significantly improving diagnostic rates and far surpassing the accuracy of traditional physician-based interpretation of imaging studies (118, 119).

Regarding disease activity assessment, studies have demonstrated that deep learning-assisted systems offer an accurate and convenient method for measuring exophthalmos in patients with thyroid-associated ophthalmopathy using facial photographs. This provides a promising alternative to traditional exophthalmos measurement techniques, enhancing the accessibility of reliable exophthalmos assessment in both clinical and non-specialist settings (120). Multiple studies indicate that by analyzing patient data such as eye protrusion, degree of diplopia, and inflammatory markers, artificial intelligence models can accurately assess disease activity levels, providing quantitative evidence for treatment planning (117, 121).

During the treatment monitoring phase, studies have employed wearable devices to collect ocular physiological data (such as intraocular pressure and ocular movement parameters) from patients. Real-time analysis using artificial intelligence algorithms enables dynamic monitoring of treatment efficacy. This approach facilitates the timely detection of changes in patient parameters like intraocular pressure, allowing for prompt adjustments to treatment regimens (122). Additionally, in studies involving patients receiving immunomodulatory therapy or orbital radiotherapy, artificial intelligence can analyze pre- and post-treatment imaging changes to quantitatively assess therapeutic outcomes such as reduced tissue inflammation and decreased extraocular muscle volume, thereby providing objective references for monitoring treatment progress (123).

However, the application of artificial intelligence in toxic thyroid eye disease remains in its developmental stage, with numerous challenges persisting. These include inconsistent data quality across centers, a lack of standardized, multicenter data for training models, and insufficient model generalization capabilities. Future efforts must focus on further optimization and refinement to successfully translate these advancements into clinical practice.

## 7 Conclusion

Thyroid eye disease is a complex autoimmune disease whose pathogenesis involves the interaction of multiple factors including immunity, genetics, and environment. Its diverse clinical manifestations significantly impair patients’ ocular function and quality of life. Current treatment approaches encompass general management, medical therapy, orbital radiotherapy, and surgical intervention, each with its own indications and limitations. In recent years, emerging research directions such as targeted therapy, immunomodulatory cell therapy, and artificial intelligence applications have brought new hope for the treatment and management of TED. Future efforts should focus on further elucidating the pathogenesis, exploring more effective therapeutic targets and treatment modalities, and integrating multidisciplinary diagnostic and therapeutic approaches to enhance the standard of care for TED and improve patient outcomes.
